# Broader conceptualization of remission assessed by the remission from depression questionnaire and its association with symptomatic remission: a prospective, multicenter, observational study

**DOI:** 10.1186/s12888-016-1067-3

**Published:** 2016-10-19

**Authors:** Alonso Montoya, Jeremie Lebrec, Karen Mary Keane, Irene Fregenal, Antonio Ciudad, Ángel Moríñigo, Luis Agüera-Ortiz, Irene Romera, Inmaculada Gilaberte, Mark Zimmerman

**Affiliations:** 1 Department of Medical Neurosciences, Lilly Research Laboratories, Eli Lilly Canada Inc., 3650 Danforth Avenue, Toronto, ON Canada; 2 Department of Global Statistical Sciences, Eli Lilly and Company, Werner-Reimers-Straße 2, 61352 Bad Homburg vor der Höhe, Germany; 3 Department of Clinical Development, Eli Lilly and Company, Erl Wood Manor, Windlesham, Surrey GU20 6PH UK; 4 Department of Clinical Research, Eli Lilly and Company, Avenida de la Industria, 30, 28108 Alcobendas, Madrid Spain; 5 Department of Psychiatry, University of Sevilla, Calle San Fernando, 4, 41004 Sevilla, Spain; 6 Department of Psychiatry, Hospital Universitario 12 de Octubre and CIBERSAM, Madrid, Spain; 7 Department of Psychiatry, Universidad San Pablo CEU, Madrid, Spain; 8 Department of Psychiatry and Human Behavior, Brown University School of Medicine, Rhode Island Hospital, 146 West River Street, Providence, 02904 Rhode Island USA

**Keywords:** Hamilton rating scale for depression, Major depressive disorder, Remission from depression questionnaire, Remission

## Abstract

**Background:**

Goals of treating major depressive disorder (MDD) include achieving remission and avoiding relapse. It is possible that patients may have a broader view of remission than what is captured via clinician-rated scales. This patient perspective may, in turn, have an impact on treatment outcomes.

**Methods:**

The association between a broader conceptualization of remission, based on the Remission from Depression Questionnaire (RDQ) score at baseline, and being in symptomatic remission after 6 months was evaluated in subjects (*N* = 613) with MDD in symptomatic remission at baseline (17-item Hamilton Rating Scale for Depression [HAMD-17] ≤7). Specific aspects of depression were assessed from physician and patient perspectives as secondary endpoints. A backwards selection strategy was used to statistically model remission status and determine association of factors with potential to influence remission.

**Results:**

At month 6, after adjustment for baseline HAMD-17 score, there was no association between baseline RDQ score and symptomatic remission status (HAMD-17), relapse, composite remission status, healthcare resource utilization, or quality of life. There was no association between functional impairment scores at baseline (Sheehan Disability Scale and Social and Occupational Functioning Assessment Scale) and symptomatic remission status (HAMD-17) at month 6.

**Conclusions:**

This study indicates that RDQ-constructs are independent from symptomatic remission. Symptom severity at study entry appeared to be the only significant predictor of eventual relapse during the 6-month follow-up period. However, our results also suggest that the current definition of remission that is based on symptom reduction should be further elaborated and that alternative or additional definitions should be considered in determining remission.

## Background

Current standards for treatment of major depressive disorder (MDD) recommend that achieving remission should be considered the principal goal [1]. However, in the absence of biological markers, remission from depression has proven to be an elusive construct to capture and apply. In antidepressant efficacy trials, remission is typically defined in terms of the absence of symptoms on measures such as the Hamilton Rating Scale for Depression (HAMD) [2] or Montgomery–Åsberg Depression Rating Scale (MADRS) [3]. However, exclusively symptom-based definitions may be fundamentally limited in scope [1]. While normalization of function is considered important in the definition of remission, it is not identified by the scales. Moreover, depressed patients have a more inclusive perception about what remission from a depressive episode means. The presence of features of positive mental health, a return to one’s normal self, and a return to usual level of functioning were more important to patients than the disappearance of depressive symptoms alone when they considered their remission status [4]. In fact, discordance can occur between clinicians’ and patients’ perceptions of remission [5]. In a previous study, Zimmerman and colleagues found that approximately half of patients scoring in the remission range on the HAMD did not consider themselves to be in remission [6]. In support of these observations, the use of patient-reported outcome scales in clinical trials conducted by the pharmaceutical industry has become more widespread [7].

Acknowledging that symptom-based measures do not accommodate these additional factors [3, 4], researchers have advocated a broader conceptualization of remission that includes factors beyond depressive symptoms [8, 9]. The recently developed Remission from Depression Questionnaire (RDQ) assesses remission in a broader and multidimensional way. The RDQ is patient-completed and addresses other symptoms often present in depressed patients, such as anxiety and irritability, features of positive mental health, coping ability, functioning, life satisfaction, and a general sense of well-being [10].

One of the principal goals of defining remission is to predict future morbidity. The basis for the emphasis on “treating to remission” is the consistent finding that treatment responders who meet the threshold for symptomatic remission are significantly less likely to relapse than those who do not [4]. The patient perspective on remission is broad and multidimensional, while the current definition of remission based on the 17-item Hamilton Rating Scale for Depression (HAMD-17) is focused on clinical symptoms of depression only. The implication of this different conceptualization of remission on prognosis has not been studied previously.

We describe here the results of a multicenter, observational study in depressed patients in symptomatic remission who were treated in routine clinical practice. The primary objective of the study was to determine whether RDQ score at the end of an acute treatment phase (study baseline) was associated with symptomatic remission, defined using the HAMD-17 total score at month 6. Our secondary objectives included assessment of the association of remission as measured by the RDQ with an alternative definition of relapse, and also with a composite remission status measured by a combination of clinical, functional, and quality of life criteria, healthcare resource use, and quality of life. Additionally, we assessed the association of symptomatic remission at month 6 (by HAMD-17) with patient-rated or clinician-rated baseline functioning status evaluated by Sheehan Disability Scale (SDS) or Social and Occupational Functioning Assessment Scale (SOFAS), respectively.

Our hypothesis was that in patients with MDD who are in symptomatic remission based on the HAMD (HAMD-17 ≤ 7) after the acute treatment of a depressive episode, a broad perspective of remission based on the RDQ at completion of an acute treatment phase, is associated with being in symptomatic remission at the end of the 6-month follow-up period.

## Methods

### Study design and participants

This prospective, multicenter, observational study was conducted in 613 subjects at 39 centers in Spain from June 2012 to July 2013 (first and last subject visit). The study was conducted in accordance with the ethical principles that have their origin in the Declaration of Helsinki and are consistent with Good Clinical Practice guidelines. The protocol was submitted and approved by appropriate ethical review boards. All subjects provided written informed consent before enrollment in the study.

Treatment for the current major depressive episode was solely at the discretion of the physician and was prescribed in the usual standard of care. Participation in the study did not influence payment or reimbursement for any treatment received by subjects during the study.

The study included visits at baseline and month 6 (±2 weeks). Additionally, the assessment of relapse at month 6 covered the entire 6-month period from baseline. Baseline patient characteristics including history of MDD (any prior episode of MDD before the episode referred to for inclusion in the study), MDD treatment history, chronic somatic co-morbidities, concomitant therapy, and medications/MDD treatment were summarized for the full analysis set (FAS), by month 6 depression status (by HAMD-17 remission status), and overall. Validated survey instruments were administered at each visit to assess clinical and health outcomes. Data, including electronic patient-reported outcomes, were collected electronically.

Outpatients ≥18 years of age with a diagnosis of MDD (based on the Spanish validated version 6.0 Mini-International Neuropsychiatric Interview (MINI) interview [11]), who previously went through the acute treatment phase (12 ± 2 weeks prior to baseline visit) of the current depressive episode and were in symptomatic remission, were recruited from private psychiatric medical clinics. This window (12 ± 2 weeks) was used in previous trials and was intended to capture patients in remission after completion of a recent acute treatment phase. Symptomatic remission was defined as a score of ≤7 on the HAMD-17 and the absence of a *Diagnostic and Statistical Manual of Mental Disorders*, 4th edition (DSM-IV) diagnosis of depression at baseline as assessed by the MINI.

### Outcome measures (definitions of remission, functioning, and quality of life)

The primary objective of the study was to assess the relationship between symptomatic remission evaluated by the HAM-D17 at month 6 and baseline RDQ total score.

#### Remission from Depression Questionnaire (RDQ)

The RDQ captures a broad array of domains including symptoms of depression, non-depressive symptoms (e.g., anxiety and irritability), features of positive mental health, coping ability, functioning, life satisfaction, and a general sense of well-being that patients consider important in determining their remission status. The RDQ is a reliable and valid measure [12] with 41 items and seven subscales. Items are reported on a 3-point rating scale: 0 = not at all or rarely true; 1 = sometimes true; and 2 = often or almost always true. Patients who have RDQ total score ≤27 are considered in remission, and higher item values reflect greater pathology. In this study, the RDQ score is considered to represent the patient’s perception of his or her own remission status and reflects multidimensional factors often present in depressed patients.

#### 17-item Hamilton rating scale for depression (HAMD-17)

The HAMD-17 assesses the range of symptoms most frequently observed in patients with MDD [2]. Remission is defined in terms of the absence of symptoms, with ‘asymptomatic’ operationalized as a score of ≤7. In this study, the HAMD-17 was used to assess symptomatic remission at baseline and study end (month 6 [sustained remission]).

#### The Mini-International Neuropsychiatric Interview (MINI)

The MINI is a psychiatric structured interview widely used to establish psychiatric diagnosis according to the DSM-IV. It is divided into modules corresponding to different diagnostic categories. The MINI depression module was used to establish the presence or absence of a diagnosis of depression and to indicate relapse (reported as relapse [yes/no]) at month 6 ± 2 weeks.

#### Quick Inventory of Depressive Symptomatology-Self Report (QIDS-SR)

The Quick Inventory of Depressive Symptomatology–Self Report (QIDS-SR) is a reliable and valid measure of the DSM-IV symptom criteria of MDD [13]. In this study, a score of ≤5 indicated remission (reported as yes/no).

#### Social and Occupational Functioning Assessment Scale (SOFAS)

The SOFAS indicates the level of social and occupational functioning across a continuum, ranging from a state of optimum functioning to a state of worst functional impairment, without taking symptoms into account [14]. In this study, results were reported as good/bad functioning based on a score of ≥80 indicating good functioning [15].

#### Sheehan Disability Scale (SDS)

The SDS [16] is a patient-rated scale that assesses disability by evaluating impairment in work/school, social life/leisure activities, and family life/home responsibilities. In this study, results were reported in terms of functional impairment with a total score ≥6 indicating overall functional impairment reported as overall/no overall functional impairment [17].

#### EuroQol (EQ-5D)

The EuroQol (EQ-5D), a widely used generic, health-related (disease non-specific) quality of life instrument [18], provides a utility score related to health status and impact on usual life activities. In this study, index scores using the Spanish value sets were reported [19]. Patients with an index scores of >70.71 were considered in remission (nonremission ≤70.71) [20].

### Alternative definitions of remission

Association of baseline RDQ remission status with alternative definitions of remission/relapse was evaluated. Depressive status evaluated by the MINI at month 6 was used as an indication of relapse. Alternatively, 2 combinations of outcome measures, the QIDS-SR, SDS, and EQ-5D, and the HAMD-17 and SOFAS, provided a basis for composite remission status. The composite scores assessed a wide range of attributes. Briefly, the QIDS-SR, SDS, and EQ-5D measure depressive symptoms, disability, and health-related quality of life, respectively.

Composite remission status (i.e., yes or no) was determined based on cutoffs for QIDS-SR, SDS, EQ-5D, HAMD-17, and SOFAS. Patients who met the cutoff criterion on each scale were considered as meeting the criteria for composite remission. Patients who did not meet the cutoff or missed information on any of the 3 scales (QIDS-SR, SDS, and EQ-5D) were considered as not meeting the composite remission criterion.

#### Functioning

The association of symptomatic remission at month 6 (according to HAMD-17) with baseline functioning status was assessed as patient-rated by the SDS or clinician-rated by the SOFAS.

#### Resource utilization

The number and percentage of subjects using healthcare resources, stratified by RDQ baseline status, were analyzed in a descriptive manner. Moreover, the relationship between resource utilization and remission status was explored by means of logistic regression analyses with resource utilization outcomes dichotomized (Number of Visits >0 or not >0) for analysis.

Healthcare resource utilization, at baseline (utilization in previous 30 days) and month 6 (all utilization in the 6 months between baseline and month 6), was based on the following questions:

1. Has the subject used any additional healthcare resources?
2. How many emergency room or equivalent facility visits for psychiatric illness have occurred?
3. How many emergency room or equivalent facility visits for nonpsychiatric illness have occurred?
4. How many outpatient visits to other physicians (not psychiatrists) have occurred?
5. How many psychiatric visits have occurred?
6. How many hospitalizations for any reason have occurred?
7. Is the subject currently on sick leave due to depression?

### Safety analyses

No safety information was collected for this study. Investigators treated adverse events according to pharmacovigilance procedures.

### Statistical methods

#### Study population

The FAS included all subjects who provided consent to release information and fulfilled study entry criteria.

#### Determination of sample size

It was determined that a sample size of 499 observations would achieve 80 % power at a 0.05 significance level to detect a change in the probability of relapse from the value of 0.15 at the mean of RDQ to 0.209 when RDQ score is increased to 1 standard deviation (SD) above the mean (calculation based on [21]). This change corresponds to an odds ratio of 1.5. An adjustment was made since a multiple regression of the independent variable of interest on the other independent variables in the logistic regression obtained an r^2^ of 0.25. Considering that withdrawals were expected during the study (approximately 10 %), a sample size of 550 patients was needed at baseline.

### Treatment outcome analyses

#### Clinical outcomes

Total scores and subscale scores were summarized (as applicable) for each clinical scale and questionnaire. Summary statistics by time and remission status as well as change from baseline were presented for those questionnaires for which total scores could be derived.

Binary outcomes (e.g., remission status) and continuous outcomes (e.g., change from baseline in RDQ) were modeled with respect to other factors such as baseline RDQ total score by logistic regression and linear regression, respectively. The initial models included baseline severity of depression (as determined by HAMD-17), age, gender, employment status, number of years since diagnosis, family history of depression, history of MDD, duration of treatment for current episode, and somatic comorbidities. The variable selection process began with removing the largest nonsignificant p-values (i.e., *p* > 0.05) one at a time while always retaining all other variables. The significance of the reduced model was tested using the likelihood ratio test and if significant, the previously removed variable was returned to derive the final model. The Hosmer-Lemeshow test was used to check for goodness of fit of the final logistic regression models while the final linear regression model was tested against the null model using the F-test.

To assess the association of function based on baseline scores of the SOFAS and SDS and symptomatic remission at month 6 (as determined by HAMD-17), a new model was developed that included SOFAS/SDS status at baseline (dichotomized to good functioning/bad functioning) as an independent variable. The same variable selection process was applied as for the other outcomes, however the variables severity of depression, sex, and age were forced into the model.

#### Health outcomes

Subject quality of life at baseline and 6 months (as evaluated by EQ-5D) and the association with the RDQ score at baseline was analyzed by means of an Analysis of Covariance (ANCOVA) model. The model included the change in the score as the dependent variable, the RDQ status (i.e., remission, nonremission) at baseline, the baseline value of the respective score as a covariate, as well as baseline severity of the depression (as evaluated by HAMD-17), sex, age, and other relevant baseline variables. The same variable selection process was applied as for the other outcomes.

Healthcare resource utilization variables were analyzed in a descriptive manner, stratified by binary RDQ baseline status. Moreover, the relationship between resource utilization and the subject’s RDQ remission status at baseline was explored by means of logistic regression analyses. The effect of RDQ score on month-6 outcome was assessed using the same modeling approach.

All analyses were performed using SAS 9.1.3 (or later) for Windows, SAS Institute Inc., Cary, NC, USA. No adjustment for multiple comparisons was made.

## Results

The FAS included all 613 subjects enrolled in the study. At the month-6 visit, 38 subjects had discontinued. Reasons for discontinuation after baseline (*n* = 35) included lost to follow-up (*n* = 20), site unresponsive (*n* = 12), physician decision (*n* = 1), and subject decision (*n* = 2).

The mean (± SD) age of subjects in the study was 46.6 (13.5) years and the majority were female (68.8 %). Most subjects had received university (38.5 %) or secondary education (35.6 %), were able to work (76.2 %), and were living with a family member (89.1 %). Approximately half of the subjects (49.9 %) had a family history of depression. This was not the first episode of MDD for 56.0 % (*n* = 343) of subjects. Further, 46.1 % of subjects had received prior treatment. The most frequent antidepressant drug treatment was selective serotonin reuptake inhibitors (SSRI) (60.9 %). The mean treatment period for prior episodes of MDD was 31.4 ± 40.4 months. There were no apparent differences in demographic characteristics between subjects in remission at baseline and subjects not in remission (HAMD-17) at month 6 (Table 1). The percentage of patients who were in remission at baseline according to the dichotomous definition was 75.2 %. The majority of subjects (98.1 %) who were in remission according to RDQ status at month 6 were also in remission by according to HAMD-17.

**Table 1:** Baseline characteristics for full analysis set and by remission status at month 6

	FAS	Based on HAMD-17 at Month 6	
	*N* = 613	*N* = 575	
		Remission	No Remission
		*n* = 521	*n* = 54
Age (years)			
Mean (SD)	46.6 (13.5)	46.6 (13.4)	48.6 (14.3)
	*n* (%)	*n* (%)	
Sex			
Male	191 (31.2)	166 (92.7)	13 (7.3)
Female	422 (68.8)	355 (89.6)	41 (10.4)
Education status			
No formal education	11 (1.8)	8 (72.7)	3 (27.3)
Primary education	148 (24.1)	131 (93.6)	9 (6.4)
Secondary education	218 (35.6)	176 (88.9)	22 (11.1)
University education	236 (38.5)	206 (91.2)	20 (8.8)
Living with family, partner, or friend			
Yes	546 (89.1)	464 (90.8)	49 (9.6)
No	67 (10.9)	57 (91.9)	5 (8.1)
Work status			
Yes^a^	467 (76.2)	394 (89.3)	47 (10.7)
No^b^	138 (22.5)	127 (94.8)	7 (5.2)
Other	8 (1.3)	-	-
Family history of depression			
Yes	306 (49.9)	264 (90.4)	28 (9.6)
No	307 (50.1)	257 (90.8)	26 (9.2)
Is this the first episode of MDD?			
Yes	270 (44.0)	237 (93.7)	16 (6.3)
No	343 (56.0)	284 (88.2)	38 (11.8)
Previous treatment for MDD			
n	605	515	53
Yes	279 (46.1)	231 (88.8)	29 (11.2)
No	326 (53.9)	284 (92.2)	24 (7.8)
Missing	8	1	7
Previous treatment for current MDD episode^c^			
n	613	521	54
Yes	589 (96.1)	501 (90.3)	54 (9.7)
No	24 (3.9)	20 (100)	0 (0.0)
Missing	0	0	0
Previous treatment duration–current MDD episode^c^			
*n*	589	501	54
Mean (SD), months	5.7 (12.07)	5.9 (12.95)	5.3 (4.84)
Missing	0		
RDQ (Total Score)			
*n*	571	512	53
Mean (SD)	17.5 (14.6)	12.0 (11.1)	43.4 (16.0)
HAMD-17 (Total Score)			
*n*	575	521	54
Mean (SD)	4.0 (2.0)	2.4 (1.9)	12.9 (4.6)
RDQ remission at baseline			
*n*	613	517	54
Remission	461	396 (90.4)	42 (9.6)
Nonremission	148	121 (91.0)	12 (9.0)
Missing	4	4	0
RDQ remission at month 6			
*n*	575	512	53
Remission	471	462 (98.1)	9 (1.9)
Nonremission	94	50 (53.2)	44 (46.8)
Missing	10	9	1

Abbreviations: *FAS* full analysis set, *HAMD-17* 17-item Hamilton Rating Scale for Depression, *MDD* major depressive disorder, *n* number of patients with a nonmissing observation, *RDQ* remission from depression questionnaire, *SD* standard deviation

^a^Yes = part-time, self-employed, student, house-keeping, volunteer, full-time

^b^No = unable to work, unemployed, retired

^c^Current episode refers to the index MDD episode that led to the patient’s entry into the study

For the current MDD episode, 96.1 % of subjects had received prior treatment, 77.3 % were treated at baseline and 61.5 % were treated at month 6, respectively. Treatment patterns were similar across visits, with antidepressants being the most frequently administered: SSRIs (51.4 % at baseline), serotonin norepinephrine reuptake inhibitors (SNRIs) (34.3 % at baseline) and tricyclic antidepressants (3.2 % at baseline) (Table 2).

**Table 2:** Treatment history for major depressive disorder

Treatment for current MDD episode		
Prior to visit^a^	Baseline	Month 6
	*N* = 613	*N* = 613
Any treatment (*n*)	589 (96.1 %)	NA
Psychotherapy	29 (4.9 %)	NA
Tricyclic antidepressants	19 (3.2 %)	NA
SSRI	303 (51.4 %)	NA
SNRI	202 (34.3 %)	NA
Antipsychotics	0 (0.0 %)	NA
Mood stabilizers	4 (0.7 %)	NA
Light therapy, ECT, or herbal remedies	0	NA
Other	32 (5.4 %)	NA
At visit^b^	Baseline	Month 6
Any treatment (*n*)	474 (77.3 %)	377 (61.5 %)
Psychotherapy	22 (4.6 %)	14 (3.7 %)
Tricyclic antidepressants	15 (3.2 %)	15 (4.0 %)
SSRI	225 (47.5 %)	168 (44.6 %)
SNRI	182 (38.4 %)	154 (40.8 %)
Antipsychotics	2 (0.4 %)	2 (0.5 %)
Mood stabilizers	4 (0.8 %)	6 (1.6 %)
Light therapy, ECT, or herbal remedies	0	0
Other	24 (5.1 %)	18 (4.8 %)

Abbreviations: *ECT*, electroconvulsive therapy, *MDD* major depressive disorder, *N* number of patients involved in the summary, *n* number of patients in the given category, *NA* not applicable, *SNRI* serotonin norepinephrine reuptake inhibitors, *SSRI* selective serotonin reuptake inhibitors

^a^Apart from the row, ‘Any treatment’, all other percentages in ‘Prior to visit’ section use. *n* = 589 (n of patients with any treatment) as denominator

^b^Apart from the row, ‘Any treatment’, all other percentages in ‘At visit’ section use. *n* = 474 (n of patients with any treatment) as denominator

### Association of baseline RDQ with remission by HAMD-17 at month 6

**Fig. 1:**
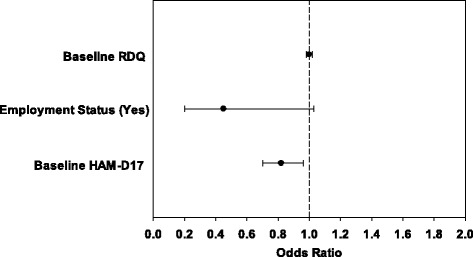
Association of baseline remission from depression questionnaire with remission status by HAMD-17 at Month 6. Odds ratios and 95 % confidence intervals are shown. The baseline HAMD-17 variable reflects a single point increase in the baseline HAMD-17 score. Abbreviations: HAMD-17, 17-item Hamilton Rating Scale for Depression; RDQ, Remission from Depression Questionnaire

The multivariate logistic regression model to evaluate the association of RDQ status at baseline (as a continuous or dichotomous variable) with sustained remission showed that an increase in baseline HAMD-17 score of 1 unit significantly decreased the odds of the patient being in remission by 18 %, ([odds ratio [OR] 0.82, 95 % confidence interval [CI] 0.70 to 0.96, *p* = 0.017) for the model with RDQ status as a continuous variable (Fig. 1) and by 19 %, (OR 0.81, 95 % CI 0.70 to 0.95, *p* = 0.009]) for the model with RDQ status as a dichotomous variable (Table 3). The final selected model only included employment status, HAMD-17, and RDQ. Increasing RDQ by 1 unit did not change the odds of the patient being in sustained remission (OR 1.0, 95 % CI 0.98 to 1.02, *p* = 0.925). Remission for dichotomous RDQ at baseline decreased the odds of the patient being in sustained remission by 11 % (OR 0.89, 95 % CI 0.59 to 1.33), although the decrease was not significant (*p* = 0.568).

**Table 3:** Logistic regression analyses

Dependent variable categorical outcome at month 6	Independent variable score at baseline	*p*-value	Level/Unit	Odds Ratio	95 % CI
Remission					
HAMD-17	RDQ	0.439	Remission	0.76	0.38, 1.52
	HAMD-17	0.009	1 unit	0.81	0.70, 0.95
Relapse					
MINI	RDQ	0.071	1 unit	0.98	0.96, 1.00
	HAMD-17	0.007	1 unit	1.21	1.05, 1.39
Composite remission status					
QIDS-SR + SDS + EQ-5D	RDQ	0.553	1 unit	0.99	0.96, 1.02
HAMD-17 + SOFAS	RDQ	0.374	1 unit	0.99	0.97, 1.01
Functioning					
HAMD-17	SDS	0.789	No impairment	1.09	0.57, 2.09
	HAMD-17	0.017	1 unit	0.81	0.69, 0.96
HAMD-17	SOFAS	0.917	Good functioning	1.03	0.55, 1.95
	HAMD-17	0.024	1 unit	0.83	0.71, 0.98
Healthcare resource utilization^a^					
Healthcare resources	RDQ	0.568	Remission	0.89	0.59, 1.33

Abbreviations: *CI* confidence interval, *EQ-5D* EuroQol, *HAMD-17* 17-item Hamilton Rating Scale for Depression, *MINI* mini-International neuropsychiatric interview, *QIDS-SR* quick inventory of depressive symptomatology-self report, *RDQ* remission from depression questionnaire, *SDS* Sheehan disability scale, *SOFAS* social and occupational functioning assessment scale

^a^Healthcare Resource Utilization was coded as a categorical outcome with at least one visit receiving a ‘Yes’

After adjustment of HAMD-17 score at baseline (the scale used to measure baseline remission), there was no association between baseline RDQ score as a continuous or dichotomous variable and remission status (according to HAMD-17) at month 6.

### Alternative definitions of remission

#### Association of baseline RDQ score with MINI

The association of RDQ score at baseline with relapse at month 6 measured by MINI, modeled by logistic regression, showed an increase of 1 unit in baseline RDQ was associated with a nonsignificant decrease in the odds of relapse by 2 % (OR 0.98, 95 % CI 0.96 to 1.00, *p* = 0.071). In contrast, an increase in 1 unit in HAMD-17 at baseline corresponded to a significant increase of 21 % in the odds of relapse at 6 months (OR 1.21, 95 % CI 1.05 to 1.39, *p* = 0.007). Sex, age, and employment were significant factors (*p* < 0.05).

#### Association of baseline RDQ with composite remission status

Logistic regression models showed no association of RDQ at baseline with composite remission status (i.e., yes/no) at month 6 measured by QIDS-SR, SDS, and EQ-5D or by HAMD-17 and SOFAS. An increase in RDQ score of 1 unit at baseline was associated with a nonsignificant decrease of 1 % in the odds of being in sustained remission for both composite scores (QIDS-SR, SDS, and EQ-5D = OR 0.99, 95 % CI 0.96 to 1.02, *p* = 0.553; HAMD-17 and SOFAS = OR 0.99, 95 % CI 0.97 to 1.01, *p* = 0.374).

### Patient’s functioning

#### Association of baseline functioning (SDS, SOFAS) with remission per HAMD-17 at month 6

There was no association between remission scores (HAMD-17 ≤ 7) at 6 months and both functional disability scores (SDS) and good functioning scores (SOFAS) at baseline. No overall functional disability at baseline corresponded to a 9 % nonsignificant increase in the odds of being in remission at 6 months compared to overall functional disability at baseline (OR 1.09, 95 % CI 0.57 to 2.09, *p* = 0.789).

Similarly, good functioning at baseline was associated with a 3 % nonsignificant increase in the odds of being in remission at 6 months compared to bad functioning at baseline (OR 1.03, 95 % CI 0.55 to 1.95, *p* = 0.917).

### Healthcare resource utilization and quality of life

Healthcare resource utilization was analyzed in a descriptive manner stratified by binary RDQ status (i.e., remission, nonremission) (Table 4). Use of additional healthcare resources was similar at baseline for subjects in remission (22.1 %) and subjects not in remission (27.7 %). At month 6, healthcare resource utilization increased to 35.2 % for subjects in sustained remission and 38.3 % for subjects not in sustained remission. Analysis by ANCOVA showed that change from baseline EQ-5D score was 0.023 points less for subjects in remission by RDQ than for subjects not in remission (coefficient; −0.023, 95 % CI −0.048 to 0.003, *p* = 0.077), indicating no relationship. A 1-unit higher baseline EQ-5D score was associated with a 0.26 higher EQ-5D score at month 6 (*p* < 0.001, 95 % CI 0.18 to 0.34). Baseline HAMD-17 score was not a significant factor (*p* = 0.241) in the analysis of EQ-5D.

**Table 4:** Healthcare resource utilization (Baseline) by remission from depression questionnaire baseline status

	RDQ baseline status			
	Baseline		Month 6	
	Remission	Non-remission	Remission	Non-remission
	*N* = 461	*N* = 148	*N* = 461	*N* = 148
Use of additional healthcare resources				
*n*	461	148	438	133
Yes, *n* (%)	102 (22.1)	41 (27.7)	154 (35.2)	51 (38.3)
No, *n* (%)	359 (77.9)	107 (72.3)	284 (64.8)	82 (61.7)
Visits to ER or equivalent for psychiatric illness				
*n*	102	41	154	51
Mean (SD)	0.1 (0.48)	0.1 (0.36)	0.1 (0.30)	0.1 (0.31)
Visits to ER or equivalent for nonpsychiatric illness				
*n*	102	41	154	51
Mean (SD)	0.1 (0.46)	0.4 (0.63)	0.1 (0.52)	0.4 (0.80)
Visits to physicians other than psychiatrists				
*n*	102	41	154	51
Mean (SD)	1.0 (1.10)	1.5 (1.25)	2.6 (3.30)	3.1 (3.94)
Psychiatric visits				
*n*	102	41	154	51
Mean (SD)	0.8 (0.97)	1.0 (1.16)	2.2 (2.10)	2.7 (2.03)
Hospitalizations for any reason				
*n*	102	41	154	51
Mean (SD)	0.0 (0.10)	0.0 (0.22)	0.1 (0.30)	0.1 (0.27)
Currently on sick leave due to depression				
*n*	461	148	438	133
Yes, *n* (%)	5 (1.1)	7 (4.7)	3 (0.7)	4 (3.0)
No, *n* (%)	456 (98.9)	141 (95.3)	435 (99.3)	129 (97.0)

Abbreviations: *ER* emergency room, *n* number of patients with a non-missing observation, *RDQ* remission from depression questionnaire, *SD* standard deviation

Denominators for percentages are based on nonmissing data

## Discussion

In the present study, the logistic regression models used to assess the relationship of remission defined by several outcome measures indicated no association between baseline RDQ score and remission status (according to HAMD-17) at 6 months; no association between baseline RDQ score and composite remission status based on the combination of QIDS-SR, SDS, and EQ-5D evaluations or HAMD-17 and SOFAS evaluations, healthcare resource utilization, level of quality of life (according to EQ-5D) or relapse (according to MINI). Additionally, there was no association between functional impairment scores at baseline (according to SDS), good functioning at baseline (according to SOFAS) and remission status (according to HAMD-17) at 6 months.

Previous research found that remission from MDD means more than the absence of depressive symptoms. Zimmerman et al. [8] established that severity of depressive symptoms, functional impairment, and quality of life were significantly correlated with remission status and that each of these variables was a significant, independent predictor of depressed patients’ subjective evaluations of their remission status. Acknowledging that symptom-based measures like the HAMD-17 do not accommodate these additional factors, [3, 4], researchers have advocated a broader conceptualization of remission to assess factors beyond depressive [8, 9]. Outcome measures like the RDQ more broadly assess the patient’s return to normalcy and more adequately reflect the patient’s perspective on his or her own remission status. Clinicians’ and patients’ perceptions of remission may disagree when patients score in the remission range on the HAMD but do not consider themselves in remission, [22] or conversely when patients fail to meet the HAMD definition of remission but consider themselves in remission even though they continue to experience mild symptom levels [22]. Additionally, several studies have reported that a cutoff score of ≤7 may be too liberal to consider that a patient with MDD is truly in remission [23, 24]. The findings raise caution in relying exclusively on symptom-based definitions of remission to guide treatment decision-making in clinical practice. Relying on global measures of functional impairment does little to reduce both symptoms and impairment because residual functional impairment may persist after symptoms remit [8]. A broader conceptual framework for remission will likely capture a fuller spectrum of the symptoms that patients use to make decisions regarding treatment initiation and discontinuation. Measuring both functional and symptom outcomes may help clinicians understand and treat depression.

The fact that MDD is etiologically [25] and symptomatically [26] heterogeneous makes it difficult to validate any definition of remission. The threshold used to define remission should identify a comparatively homogeneous group of patients with regard to current and future morbidity, however, patients whose MDD is in remission are heterogeneous in clinical status. Classifying them according to cutoff scores (i.e., remitters, nonremitters) on symptom-rating scales identifies groups who differ in their risk of relapse and levels of current psychosocial impairment. Patients who achieve remission have less concurrent psychosocial impairment and lower likelihood of relapse than patients who do not achieve remission [27].

The results of the present study support observations that functional remission is not always concurrent with symptomatic remission [15, 28]. Once baseline symptoms level was taken into account, there was no association between the odds of sustained remission with baseline scores for functional disability based on SDS or good functioning based on SOFAS.

In light of the lack of association between functional and symptomatic remission observed in this study, the authors turned to the RDQ subscale results in the literature for possible explanations of the overall study findings. While the RDQ was found to be as sensitive to change as the QIDS and HAMD [12] and the effect size of the total score of the scales nearly identical, there was variability in the effect sizes among the RDQ subscales. The coping and functioning subscales had lower effect sizes than the depression symptoms subscale and changes in these subscales were the least highly correlated with changes in HAMD scores. These findings can be interpreted to mean that these dimensions change more slowly than others and as the study duration was 6-months, the maximal benefit of treatment may not yet have been achieved. Another possible explanation is that coping and functioning are more resistant to improvement than other depression symptoms; therefore, at the end of the study, they may have reached their maximal improvement, and the less robust changes reflect a less complete, but more accurate, level of improvement [12].

Lack of association between RDQ and HAMD in our study may reflect that a multifactorial definition may not be more valid than a symptom-based remission definition because symptom improvement accounts for such a large portion of the variance in determining remission status that the assessment of the other domains does not improve validity. Using an instrument as RDQ to broadly evaluate domains that depressed patients consider important in determining remission, we are in fact capturing or measuring something different than what is evaluated by the symptom-based HAMD. Lack of association between RDQ and HAMD may be explained by the discordance which can occur between clinicians’ and patients’ perceptions of remission. A lack of correlation between the QIDS, a patient-reported measure, and the HAMD has also been observed [29].

Although, after adjustment for baseline HAMD, an association between baseline RDQ and HAMD at month 6 was not observed in this particular study, the clinical use of an instrument (such as the RDQ or a future instrument) that broadly evaluates domains which may capture a more complete view of a patient’s functioning and quality of life or improves the prediction of which patients are likely to have a recurrence or longer time to recurrence of depression may be beneficial to the field. Perhaps the use of a symptomatic endpoint (remission at 6 months) to assess the impact of the value of a more complex and broad measure that includes a patient’s point of view, such as the RDQ, did not totally address the impact on the prognosis of remission. In support of this, symptom reduction or even resolution is associated with improvement, but not necessarily with a full normalization of function or quality of life, and remission of a patient is neither rapid nor complete even when patients meet criteria for being in symptomatic remission [6].

### Limitations

Certain limitations to this study must be noted. First, because an observational study design was used, patients were not randomized to RDQ-remission status groups and selection bias may have influenced the results. Additionally, the relatively short follow-up may not reflect the full course of the illness. More relapses may have been observed during an extended observation period. Lastly, the origin of the sample (i.e., patients recruited from private medical clinics), which may explain the relatively high educational level of the sample, constitutes a relative bias. Perhaps a more heterogeneous sample might have influenced the results of the study.

## Conclusion

In summary, the results of this study indicate that RDQ-constructs are independent from remission. The RDQ is a multidimensional measure with inherent variability on its dimensions in terms of effect size, susceptibility to change or improvement, etc. To determine the outcome of treatment, clinicians should assess, in addition to symptom resolution, factors that patients consider important in determining remission.

## Abbreviations

ANCOVA: Analysis of covariance
CI: Confidence interval
DSM-IV: Diagnostic and statistical manual of mental disorders, 4th edition
EQ-5D: EuroQol
FAS: Full analysis set
HAMD: Hamilton rating scale for depression
HAMD-17: 17-item hamilton rating scale for depression
MADRS: Montgomery–åsberg depression rating scale
MDD: Major depressive disorder
MINI: Mini-International neuropsychiatric interview
OR: Odds ratio
QIDS-SR: Quick inventory of depressive symptomatology-self report
RDQ: Remission from depression questionnaire
SD: Standard deviation
SDS: Sheehan disability scale
SNRI: Serotonin norepinephrine reuptake inhibitors
SOFAS: Social and occupational functioning assessment scale
SSRI: Serotonin reuptake inhibitors
